# Molecular Mechanism Underlying Resistance Variation to the Novel Agrochemical Quinofumelin in *Fusarium graminearum*

**DOI:** 10.1111/mpp.70261

**Published:** 2026-04-12

**Authors:** Xiaoru Yin, Xinlong Gao, Xudong Liu, Ziyang Zhang, Qian Xiu, Fuhao Ren, Jie Zhang, Mingguo Zhou, Yabing Duan

**Affiliations:** ^1^ State Key Laboratory of Agricultural and Forestry Biosecurity, College of Plant Protection Nanjing Agricultural University Nanjing China

**Keywords:** action mode, dihydroorotate dehydrogenase, *Fusarium graminearum*, quinofumelin, resistance mechanism

## Abstract

Quinofumelin, a quinoline‐based fungicide, demonstrated potent antifungal activity against *Fusarium graminearum*, the pathogen responsible for Fusarium head blight (FHB) in wheat. Previously, we elucidated that quinofumelin targets dihydroorotate dehydrogenase (DHODH), an indispensable enzyme in the de novo pyrimidine biosynthesis pathway. To explore its resistance mechanism, five FgDHODHII site‐directed mutants with distinct genotypes were generated using the homologous recombination strategy. Sensitivity assays identified varying resistance levels: FgDHODHII‐A94V remained sensitive to quinofumelin, FgDHODHII‐D155T conferred low‐level resistance, FgDHODHII‐N281A conferred moderate‐level resistance, and FgDHODHII‐V179E/V179D conferred high‐level resistance. Quinofumelin inhibition was reversed by the addition of uridine monophosphate (UMP), uridine or uracil. Although the mutants did not exhibit growth defects, quinofumelin‐resistant mutants displayed reduced sporulation and virulence. Quinofumelin showed cross‐resistance to ipflufenoquin, but no cross‐resistance to other common fungicides (phenamacril, carbendazim, tebuconazole, pydiflumetofen, prothioconazole, fluopyram, benzovindiflupyr, pyraclostrobin and azoxystrobin). Molecular docking, molecular dynamics (MD) simulations and microscale thermophoresis (MST) revealed that D155T, V179E, V179D and N281A mutations altered the binding mode of quinofumelin, resulting in diminished binding affinity. This study provides key insights into its mode of action and resistance mechanism of quinofumelin, facilitating its application in sustainable FHB management.

## Graphical abstract

The D155T, V179E, V179D and N281A mutations in FgDHODHII altered the binding mode of quinofumelin, resulting in diminished affinity and a negative correlation between binding affinity and the resistance level of these mutants.

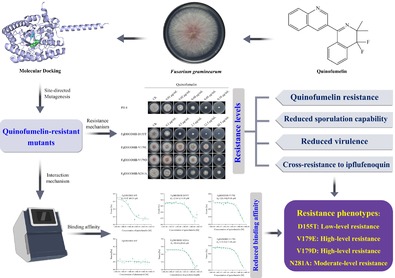

## 1 Introduction

Crop diseases severely impact agricultural yield and quality, causing significant economic losses and threatening food security (Savary et al. 2019). Fungicides are critical tools for managing plant diseases; however, their overuse leads to the development and spread of pathogen resistance, ultimately undermining control efficacy (Lucas et al. 2015; Steinberg and Gurr 2020). Therefore, elucidating the molecular mechanisms of fungicide resistance is of great significance for formulating evidence‐based resistance management strategies and provides a theoretical foundation for fungicide registration and review, as well as for the development of novel fungicides.

The genetic variation of target genes represents a primary mechanism by which plant pathogens develop resistance to fungicides, with such variations typically occurring at key amino acid residues involved in fungicide binding. For instance, in the *Cytb* gene, which is the target of quinone outside inhibitors (QoIs) such as azoxystrobin, mutations including F129L, G137R or G143S/A significantly reduce the binding affinity of the fungicide, conferring moderate to high‐level resistance to this class of fungicides (Klosowski et al. 2016; Li et al. 2022; Mao, Qiu, et al. 2024; Zhang et al. 2024). In the case of demethylation inhibitors (DMIs) such as itraconazole, mutations in the *CYP51* gene, such as Y136F, G443S, G461S, R464K, confer resistance to these fungicides (Chen et al. 2012; Mao, Meng, et al. 2024; Zhao et al. 2022). Similarly, mutations in succinate dehydrogenase (SDH) subunits, including SDHB‐P225L/T/F, SDHB‐N230I, SDHB‐H239Y/L, SDHB‐H248L, SDHB‐H272Y/R/L/V, SDHC‐A77V, SDHC‐A83V, SDHC‐G37S, SDHC‐R86K or SDHD‐H132R, are associated with resistance to succinate dehydrogenase inhibitors (SDHIs) (Hawkins and Fraaije 2018; Li et al. 2025; Yin et al. 2024; Zhang et al. 2025).

Quinofumelin (CAS: 861647‐84‐9), a novel quinoline fungicide developed by Mitsui Chemicals Co. Ltd., is classified as Fungicide Resistance Action Committee (FRAC) Group 52 based on biological characteristics (https://www.frac.info/). It exhibits strong antifungal activity against a variety of important crop pathogens, including *Fusarium graminearum*, *Pyricularia oryzae* and *Sclerotinia sclerotiorum*, and shows no cross‐resistance with existing fungicides (Higashimura et al. 2022; Tao et al. 2021; Xiu et al. 2021).

Dihydroorotate dehydrogenase (DHODH) is the fourth key enzyme in the pyrimidine biosynthesis pathway, catalysing the conversion of dihydroorotate to orotate, and is primarily localized on the inner mitochondrial membrane (Löffler et al. 2020). Pyrimidines are essential metabolites for rapidly proliferating cells such as fungi, parasites and other pathogens, as well as tumour and immune cells. Therefore, DHODH has become an important target in the medical field for the development of anti‐infective, anti‐tumour and immunomodulatory drugs (Boschi et al. 2019; Boukalova et al. 2020; Reis et al. 2017; Singh et al. 2017). In agricultural research, DHODH has been confirmed as a molecular target for herbicide development (Duke 2023). Previous research by our group found that quinofumelin targets the DHODH of *F. graminearum*, providing a key breakthrough in the discovery of new fungicide targets (Xiu et al. 2025). However, the molecular mechanism underlying resistance to quinofumelin in *F. graminearum* remains poorly understood.

To investigate the resistance mechanism of *F. graminearum* to quinofumelin and identify potential target sites, we employed AlphaFold3 to construct the three‐dimensional structure of FgDHODHII and conducted molecular docking studies with quinofumelin. We analysed the conservation of the docking sites across various fungal species. Then, using site‐directed mutagenesis, we substituted key amino acids and examined the molecular mechanism of quinofumelin resistance through sensitivity assays, molecular docking, molecular dynamics (MD) simulations and microscale thermophoresis (MST) experiments. This study aims to enhance our understanding of the regulatory mechanisms underlying FgDHODHII‐mediated sensitivity of *F. graminearum* to quinofumelin and offer critical insights for the design of novel DHODH inhibitors.

## 2 Results

### 2.1 Identification of Key Amino Acids in FgDHODHII Potentially Involved in Quinofumelin Sensitivity

**FIGURE 1:**
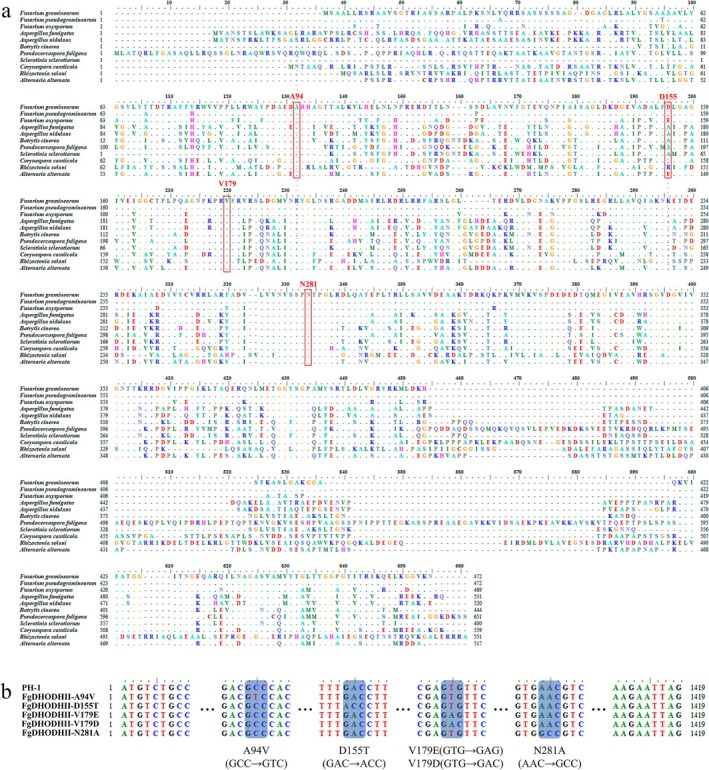
Alignment analysis of DHODHII sequences. (a) Alignment analysis of the amino acid sequences of DHODHII across 11 fungal species. The amino acid sequence of DHODHII from *Fusarium graminearum* (XP_011328027), *F. pseudograminearum* (XP_009255676), *F. oxysporum* (KAG7415722), *Aspergillus fumigatus* (KAK9596360), *A. nidulans* (AAA86932), *Botrytis cinerea* (XP_024553432), *Pseudocercospora fuligena* (KAF7189329), *Sclerotinia sclerotiorum* (XP_001585164), *Corynespora cassiicola* (PSN63766), *Rhizoctonia solani* (EUC60862) and *Alternaria alternata* (XP_018389332) were accessed in the NCBI database. (b) Amino acid mutations generated in FgDHODHII.

The protein database prediction results obtained using UniProt indicate that the residues 124–467 of FgDHODHII form a functional domain. The docking analysis of FgDHODHII with the quinofumelin molecule revealed potential binding sites at positions 72, 94, 129, 155, 179, 281, 376 and 439. Notably, the amino acids at positions 94, 155, 179 and 281 are highly conserved in various fungal species (Figure 1a). We introduced targeted point mutations in the FgDHODHII nucleotide sequence, resulting in the following amino acid substitutions: alanine to valine at position 94, aspartic acid to threonine at position 155, valine to glutamic acid or aspartic acid at position 179 and aspartic acid to threonine at position 281 (Figure 1b).

### 2.2 The D155T, V179E, V179D and N281A Mutations Confer Resistance to Quinofumelin

**FIGURE 2:**
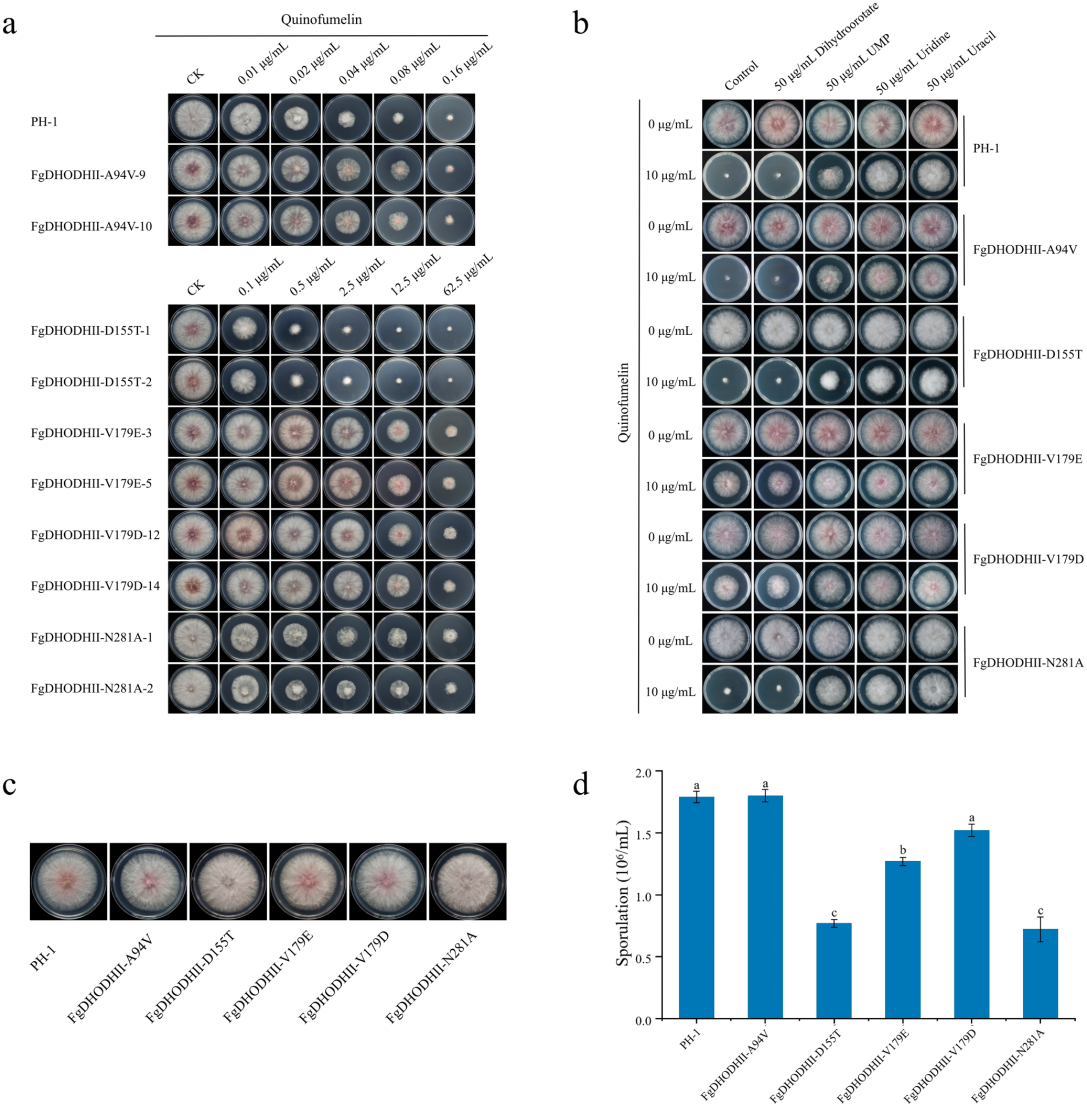
Biological fitness of site‐directed mutants in FgDHODHII. (a) Inhibitory effects of quinofumelin at varying concentrations on mycelial growth of *Fusarium graminearum* PH‐1 and the site‐directed mutants. (b) Recovery assay of PH‐1 and site‐directed mutants on potato dextrose agar plates. Mycelial growth recovery was evaluated by supplementing plates containing 10 μg/mL quinofumelin with 50 μg/mL dihydroorotate, UMP, uridine or uracil. (c) Comparison of mycelial growth rate between site‐directed mutants and PH‐1. (d) Assessment of sporulation capacity between site‐directed mutants and PH‐1. Data represent the means ± standard deviation of three independent replicates. Bars labelled with different letters indicate statistically significant differences between the site‐directed mutants and PH‐1 as determined by Fisher's least significant difference (LSD) test (*p* < 0.05).

The sensitivity to quinofumelin of 10 site‐directed mutants was evaluated using the mycelial growth inhibition method. Resistance factor (RF, RF = EC_50_ of resistant strain/EC_50_ of sensitive strain) values were used to evaluate sensitivity levels. The mutants FgDHODHII‐A94V‐9 and FgDHODHII‐A94V‐10 showed EC_50_ values of 0.04–0.06 μg/mL (RF < 5, sensitive); FgDHODHII‐D155T‐1 and FgDHODHII‐D155T‐2 showed EC_50_ values of 0.31–0.35 μg/mL (5 ≤ RF < 20, low‐level resistance); FgDHODHII‐N281A‐1 and FgDHODHII‐N281A‐2 exhibited EC_50_ values of 1.75–2.23 μg/mL (20 ≤ RF < 100, moderate‐level resistance); and FgDHODHII‐V179E‐3, FgDHODHII‐V179E‐5, FgDHODHII‐V179D‐12 and FgDHODHII‐V179D‐14 displayed EC_50_ values of 14.22–21.00 μg/mL (RF ≥ 100, high‐level resistance) (Figure 2a; Table 1). The above results confirmed that FgDHODHII is the target of quinofumelin in *F. graminearum*.

**TABLE 1:** The mutation genotypes of the site‐directed mutants in FgDHODHII and their sensitivity to quinofumelin.

Strains	Mutation genotype	EC_50_ (μg/mL)	Resistance factor^a^	Phenotype^b^
PH‐1	Wild type	0.03	—	S
FgDHODHII‐A94V‐9	A94V (GCC → GTC)	0.04	1.32	S
FgDHODHII‐A94V‐10	A94V (GCC → GTC)	0.06	1.72	S
FgDHODHII‐D155T‐1	D155T (GAC → ACC)	0.31	9.26	LR
FgDHODHII‐D155T‐2	D155T (GAC → ACC)	0.35	10.57	LR
FgDHODHII‐V179E‐3	V179E (GTG → GAG)	14.22	427.16	HR
FgDHODHII‐V179E‐5	V179E (GTG → GAG)	17.69	531.23	HR
FgDHODHII‐V179D‐12	V179D (GTG → GAC)	21.00	630.63	HR
FgDHODHII‐V179D‐14	V179D (GTG → GAC)	14.88	446.92	HR
FgDHODHII‐N281A‐1	N281A (AAC → GCC)	2.23	67.01	MR
FgDHODHII‐N281A‐2	N281A (AAC → GCC)	1.75	52.67	MR

^a^ Resistance factor (RF) is the ratio of EC_50_ for the quinofumelin‐resistant mutant to that of its wild‐type parent.

^b^ Classification of resistance levels is as follows: sensitive (S), RF < 5; low‐level resistance (LR), 5 ≤ RF < 20; moderate‐level resistance (MR), 20 ≤ RF < 100; high‐level resistance (HR), RF ≥ 100.

### 2.3 Mycelial Growth of Site‐Directed Mutants Suppressed by Quinofumelin Could Be Restored by Supplementation of the Metabolites Involved in De Novo Pyrimidine Biosynthesis Pathway

The mycelial growth of PH‐1 and the site‐directed mutants was significantly inhibited on potato dextrose agar (PDA) plates containing 10 μg/mL quinofumelin (Figure 2b). To determine whether exogenous dihydroorotate, UMP, uridine or uracil could rescue the mycelial growth of the site‐directed mutants, PDA plates containing 10 μg/mL quinofumelin were further amended with these metabolites. The results showed that while the addition of 50 μg/mL exogenous dihydroorotate failed to restore mycelial growth of PH‐1 and the site‐directed mutants, the inclusion of 50 μg/mL exogenous UMP, uridine or uracil effectively counteracted the inhibitory effects of quinofumelin on mycelial growth. Despite differences in sensitivity among FgDHODHII‐D155T, FgDHODHII‐V179E, FgDHODHII‐V179D and FgDHODHII‐N281A when exposed to 10 μg/mL quinofumelin on PDA plates, similar results were observed upon supplementation with 50 μg/mL exogenous dihydroorotate, UMP, uridine or uracil (Figure 2b). When exposed to 10 μg/mL quinofumelin on PDA plates, FgDHODHII‐V179E or FgDHODHII‐V179D exhibited partial growth but still showed significant inhibition compared to the untreated control. Supplementation with 50 μg/mL exogenous UMP, uridine or uracil restored normal mycelial growth.

### 2.4 Quinofumelin Resistance Causes a Fitness Penalty in *F. graminearum*

**FIGURE 3:**
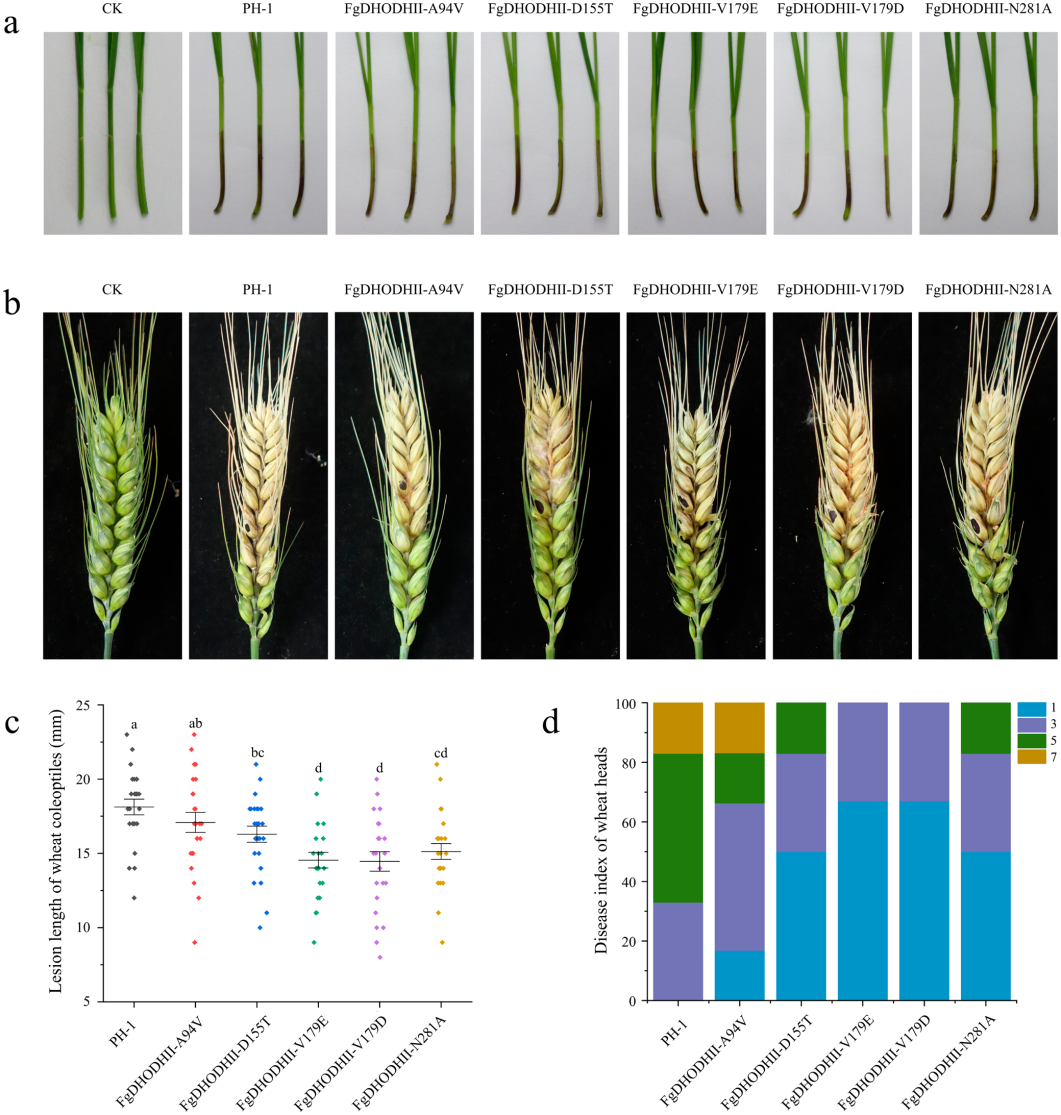
Virulence assay of the mutants carrying various mutations of FgDHODHII on wheat coleoptiles and wheat heads. (a) Lesion length in wheat coleoptiles. Each coleoptile was injected with 2 μL of conidial suspension (1 × 10^6^/mL) and incubated in a greenhouse for 7 days. (b) Assessment of virulence in wheat heads 21 days post‐inoculation. Each spike was inoculated with 10 μL of conidial suspension (1 × 10^6^/mL). The inoculation site is indicated by a black dot. Sterile water was used as the control treatment. (c) Dot plot illustrating the lesion lengths in wheat coleoptiles at 7 days post‐inoculation across different strains. Bars labelled with different letters indicate statistically significant differences between the site‐directed mutants and PH‐1 as determined by Fisher's least significant difference (LSD) test (*p* < 0.05). (d) Distribution of disease index scores in wheat heads. Disease severity classification: 1 (< 25%), 3 (25%–50%), 5 (50%–75%) and 7 (≥ 75% infected area).

There was no significant difference in mycelial growth rate among PH‐1 and the site‐directed mutants (Figure 2c). However, FgDHODHII‐D155T, FgDHODHII‐V179E, FgDHODHII‐V179D or FgDHODHII‐N281A exhibited reduced sporulation capability compared to PH‐1, whereas the sporulation capability of FgDHODHII‐A94V remained unchanged (Figure 2d). To evaluate the impact of resistance‐associated mutations on virulence, assays were conducted on wheat coleoptiles and wheat heads. In coleoptiles, the virulence of FgDHODHII‐A94V did not significantly differ from that of the wild‐type strain, while the virulence of FgDHODHII‐D155T, FgDHODHII‐V179E, FgDHODHII‐V179D or FgDHODHII‐N281A was dramatically decreased (Figure 3a,c). Similarly, the results from the virulence assays on wheat heads were consistent with those observed for wheat coleoptiles (Figure 3b,d). These findings suggest that the A94V mutation does not impair fitness, whereas the D155T, V179E, V179D and N281A mutations confer resistance at the cost of significantly reduced virulence and sporulation.

### 2.5 Cross‐Resistance Pattern

**FIGURE 4:**
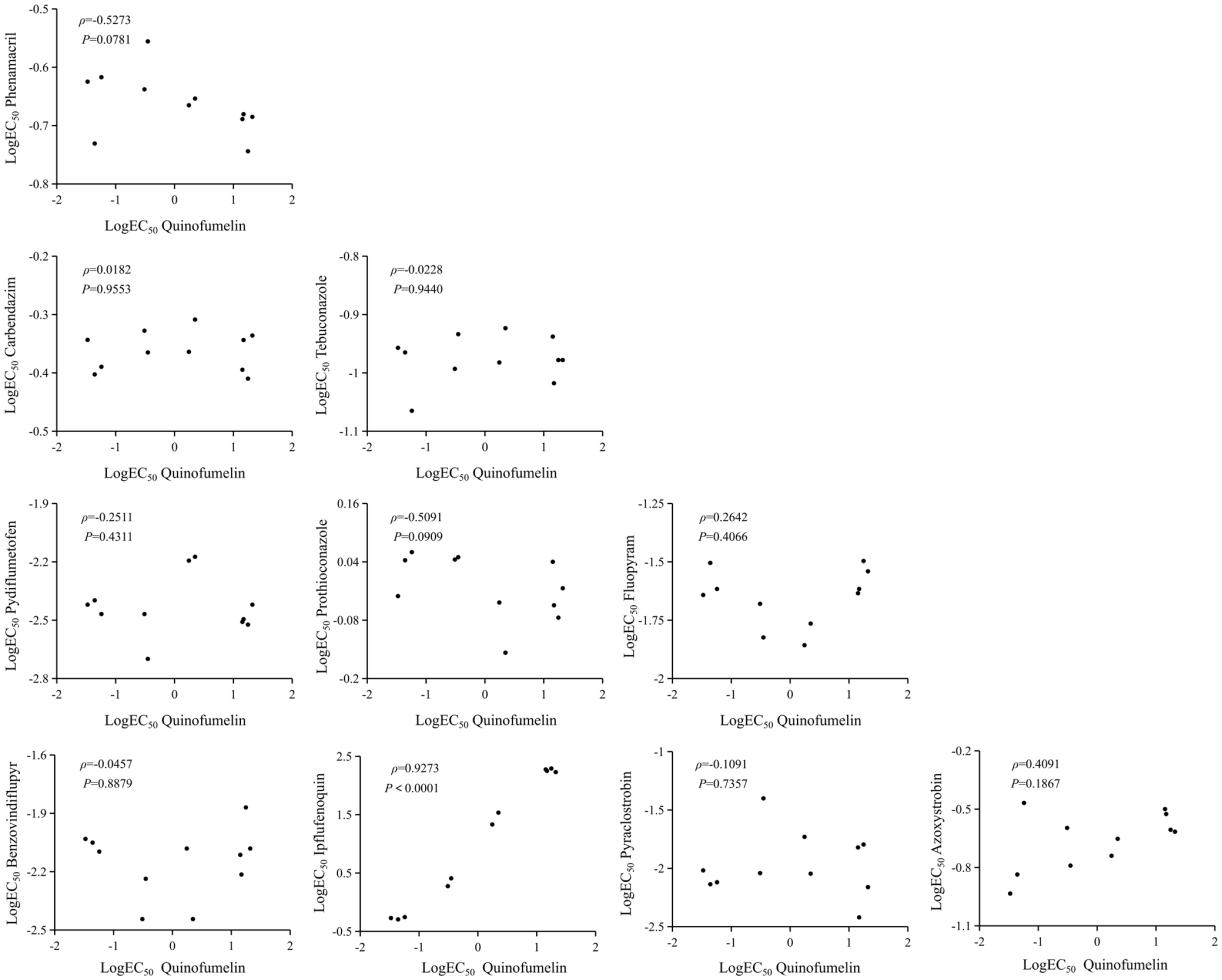
Spearman correlation tests for cross‐resistance among different fungicides. Data are logarithmic conversion of the effective concentration of 50% mycelial growth inhibition (EC_50_) values, or logEC_50_ among *Fusarium graminearum* strains for quinofumelin, phenamacril, carbendazim, tebuconazole, pydiflumetofen, prothioconazole, fluopyram, benzovindiflupyr, ipflufenoquin, pyraclostrobin and azoxystrobin. The dots represent *F. graminearum* strains used for cross‐resistance analysis. *ρ*, Spearman's rho. *p* < 0.05 indicates that the correlation difference is statistically significant.

Based on the EC_50_ values obtained from all tested strains, cross‐resistance assays were conducted between quinofumelin and 10 other fungicides. The results revealed that only ipflufenoquin (*ρ* = 0.9273) exhibited a significant positive correlation with quinofumelin, with highly similar resistance levels observed across the mutation genotypes for both fungicides (Figure 4; Table 2). In contrast, no cross‐resistance was observed between quinofumelin and phenamacril (*ρ* = −0.5273), carbendazim (*ρ* = 0.0182), tebuconazole (*ρ* = −0.0228), pydiflumetofen (*ρ* = −0.2511), prothioconazole (*ρ* = −0.5091), fluopyram (*ρ* = 0.2642), benzovindiflupyr (*ρ* = −0.0457), pyraclostrobin (*ρ* = −0.1091) or azoxystrobin (*ρ* = 0.4091) (Figure 4; Table 2). These findings suggest that ipflufenoquin may share a similar mode of action with quinofumelin.

**TABLE 2:** The sensitivity of *Fusarium graminearum* PH‐1 and the site‐directed mutants to quinofumelin and 10 other fungicides.

Strain	EC_50_ (μg/mL)										
	Quinofumelin	Phenamacril	Carbendazim	Tebuconazole	Pydiflumetofen	Prothioconazole	Fluopyram	Benzovindiflupyr	Ipflufenoquin	Pyraclostrobin	Azoxystrobin
PH‐1	0.0333	0.2373	0.4535	0.1104	0.0038	0.9323	0.0228	0.0093	0.5348	0.0096	0.1164
FgDHODHII‐A94V‐9	0.0440	0.1859	0.3957	0.1084	0.0040	1.1047	0.0313	0.0089	0.5081	0.0073	0.1459
FgDHODHII‐A94V‐10	0.0572	0.2415	0.4078	0.0861	0.0034	1.1482	0.0242	0.0080	0.5549	0.0076	0.3404
FgDHODHII‐D155T‐1	0.3084	0.2302	0.4702	0.1016	0.0034	1.1084	0.0209	0.0036	1.8850	0.0091	0.2532
FgDHODHII‐D155T‐2	0.3519	0.2782	0.4316	0.1165	0.0020	1.1209	0.0150	0.0058	2.5623	0.0398	0.1621
FgDHODHII‐V179E‐3	14.2245	0.2047	0.4031	0.1154	0.0031	1.0967	0.0232	0.0077	188.3225	0.0151	0.3165
FgDHODHII‐V179E‐5	17.6898	0.1803	0.3892	0.1052	0.0030	0.8418	0.0319	0.0135	195.7603	0.0160	0.2477
FgDHODHII‐V179D‐12	20.9999	0.2065	0.4614	0.1062	0.0038	0.9675	0.0288	0.0083	169.6468	0.0069	0.2421
FgDHODHII‐V179D‐14	14.8825	0.2087	0.4532	0.0960	0.0032	0.8926	0.0242	0.0061	178.4725	0.0038	0.2985
FgDHODHII‐N281A‐1	2.2314	0.2220	0.4914	0.1193	0.0067	0.7131	0.0172	0.0036	34.3022	0.0090	0.2226
FgDHODHII‐N281A‐2	1.7539	0.2162	0.4326	0.1042	0.0064	0.9045	0.0139	0.0083	21.5556	0.0186	0.1821

### 2.6 The D155T, V179E, V179D and N281A Mutations Reduce the Binding Affinity Between Quinofumelin and FgDHODHII

**FIGURE 5:**
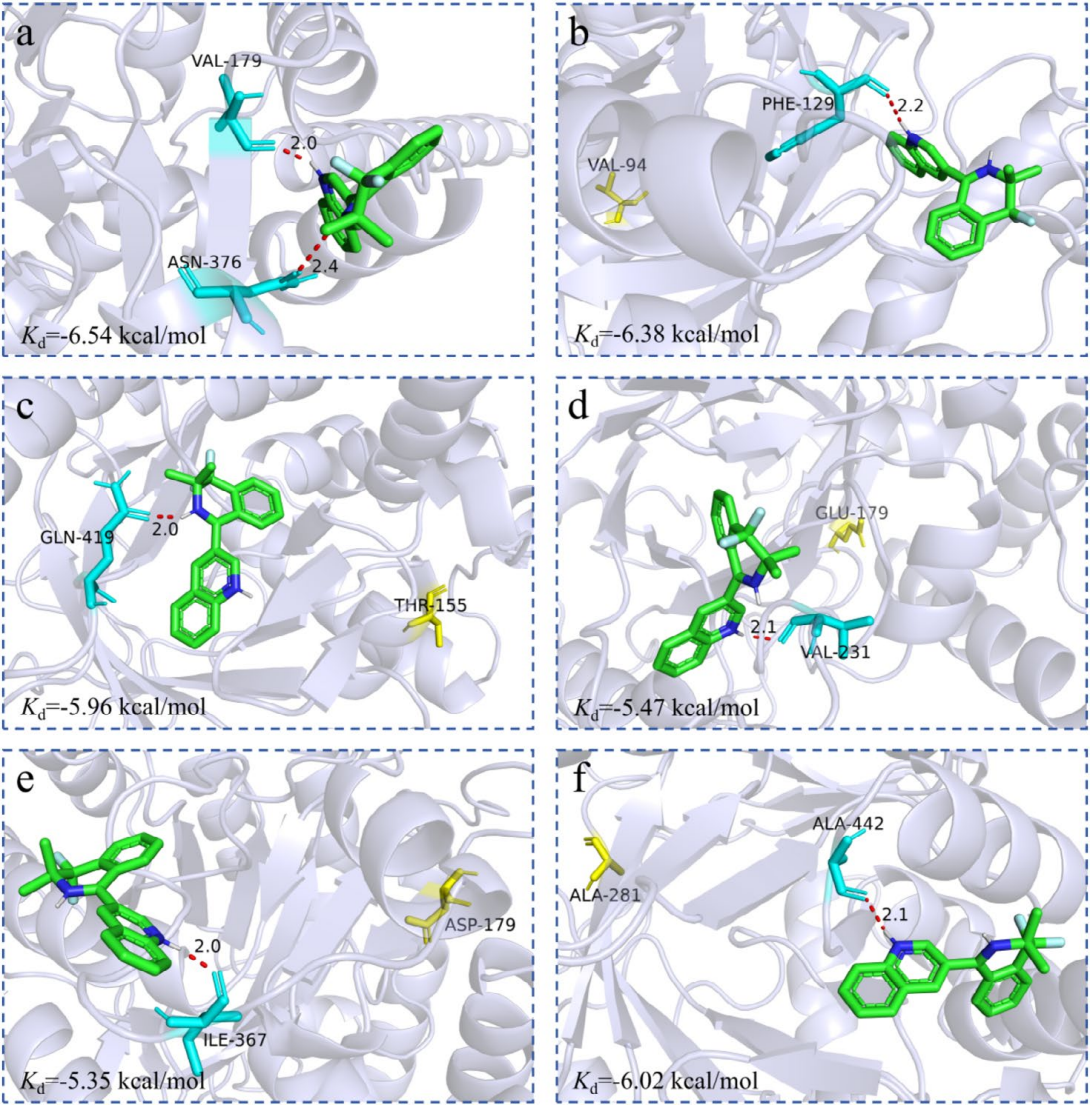
Molecular docking analysis of quinofumelin with FgDHODHII proteins. (a) Binding model of FgDHODHII‐WT protein and quinofumelin. (b–f) Binding model of FgDHODHII‐A94V, FgDHODHII‐D155T, FgDHODHII‐V179E, FgDHODHII‐V179D and FgDHODHII‐N281A proteins and quinofumelin. The FgDHODHII protein structures are displayed in cartoon format, with the key residues shown in stick representation. Mutated residues are highlighted in yellow, while hydrogen bonds between key residues and quinofumelin are indicated by red dashed lines.

The binding models of FgDHODHII wild type (WT) and five variants of FgDHODHII with quinofumelin were constructed using AutodockTools v. 1.5.7. The most favourable binding model for each variant was selected based on binding energy rankings and subsequently visualized using PyMOL v. 2.6. Molecular docking analysis revealed distinct binding patterns between quinofumelin and the five FgDHODHII variants, with noticeable variation in ligand position across mutants. The calculated binding energies were as follows: −6.54 kcal/mol for FgDHODHII‐WT with hydrogen bonds formed at F129 and N376 (Figure 5a); −6.38 kcal/mol for FgDHODHII‐A94V with one hydrogen bond at F129 (Figure 5b); −5.96 kcal/mol for FgDHODHII‐D155T with one hydrogen bond at Q419 (Figure 5c); −5.47 kcal/mol for FgDHODHII‐V179E with one hydrogen bond at V231 (Figure 5d); −5.35 kcal/mol for FgDHODHII‐V179D with one hydrogen bond at I367 (Figure 5e); and −6.02 kcal/mol for FgDHODHII‐N281A with one hydrogen bond at A442 (Figure 5f). These results indicate that the D155T, V179E, V179D and N281A mutations in FgDHODHII result in a reduced binding affinity towards quinofumelin, thus supporting a negative correlation between binding affinity and the resistance level of these mutants.

**FIGURE 6:**
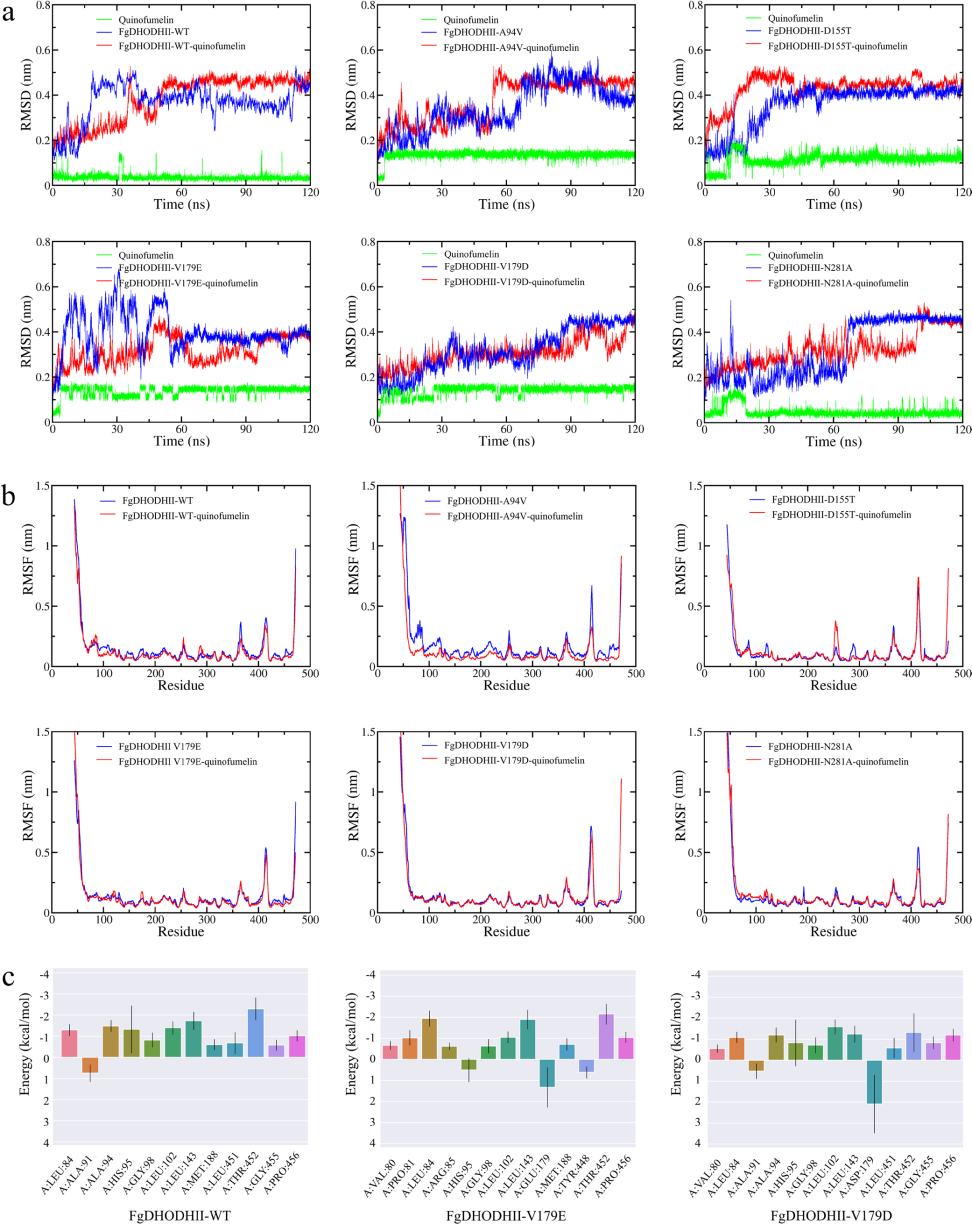
Molecular dynamics simulation of the FgDHODHII‐quinofumelin complexes. (a) The RMSD of FgDHODHII proteins and quinofumelin. (b) The RMSF of FgDHODHII proteins and quinofumelin. (c) The binding free energies of FgDHODHII protein mutants and quinofumelin.

The DHODHII–quinofumelin complexes formed by quinofumelin and five mutant forms of DHODHII were selected for 120 ns MD simulation to demonstrate their stability. The results showed that the docked complexes formed between quinofumelin and each DHODHII mutant exhibited different stable states. The RMSD values of FgDHODHII‐WT‐quinofumelin, FgDHODHII‐A94V‐quinofumelin and FgDHODHII‐D155T‐quinofumelin reached equilibration within 60 ns, whereas the remaining complexes required more than 90 ns FgDHODHII‐V179D‐quinofumelin had not equilibrated even at 120 ns (Figure 6a). Ligand RMSD traces revealed larger fluctuations and slower convergence for FgDHODHII‐V179E‐quinofumelin and FgDHODHII‐V179D‐quinofumelin compared with the other complexes. Root mean square fluctuation (RMSF) values were expected to remain below 1.5 Å for most residues, except flexible loops or terminal regions. RMSF analysis provided insights into residue‐level flexibility, showing most fluctuations below 0.6 nm, with higher deviations restricted to the N‐ and C‐terminal regions. These fluctuations were characteristic of loop and terminal segments, which did not impact the catalytic or allosteric core of DHODHII. Our results indicated that the RMSF values of FgDHODHII‐WT‐quinofumelin were less than apo FgDHODHII‐WT, suggesting that quinofumelin binding stabilized the protein structure, and the same trend was observed for A94V variant (Figure 6b). In addition, the RMSF values of the other complexes did not exhibit any appreciable reductions relative to their apo forms, implying no enhanced conformational stability (Figure 6b). Moreover, we observed that six residues (L84, H95, L102, L143, A144, T452) made notable contributions (∆G_bind < −1 kcal/mol). The V179 mutation disrupted the interactions within the binding pocket, leading to varying degrees of resistance enhancement. The binding free energies calculated by MM/PBSA were consistent with the experimentally observed trend of increased resistance (FgDHODHII‐WT: −34.87, FgDHODHII‐A94V: −34.39, FgDHODHII‐D155T: −33.98, FgDHODHII‐V179E: −29.49, FgDHODHII‐V179D: −28.65 and FgDHODHII‐N281A: −31.38 kcal/mol, respectively) (Figure 6c; Table 3).

**TABLE 3:** Binding free energies (kcal/mol) of the FgDHODHII‐quinofumelin interactions calculated by MM‐PBSA.

Proteins	∆VDWAALS	∆EEL	∆EGB	∆ESURRF	∆GGAS	∆GSOLV	∆TOTAL
FgDHODHII (wild type)	−47.61	−10.5	28.69	−5.45	−58.11	23.24	−34.87
FgDHODHII‐A94V	−47.36	−7.57	26.12	−5.58	−54.93	20.54	−34.39
FgDHODHII‐D155T	−45.06	−4.26	20.76	−5.42	−49.32	15.34	−33.98
FgDHODHII‐V179E	−43.92	−14.87	34.74	−5.44	−58.79	29.30	−29.49
FgDHODHII‐V179D	−44.15	−11.96	32.68	−5.22	−56.11	27.46	−28.65
FgDHODHII‐N281A	−40.65	−7.38	21.85	−5.20	−48.03	16.65	−31.38

**FIGURE 7:**
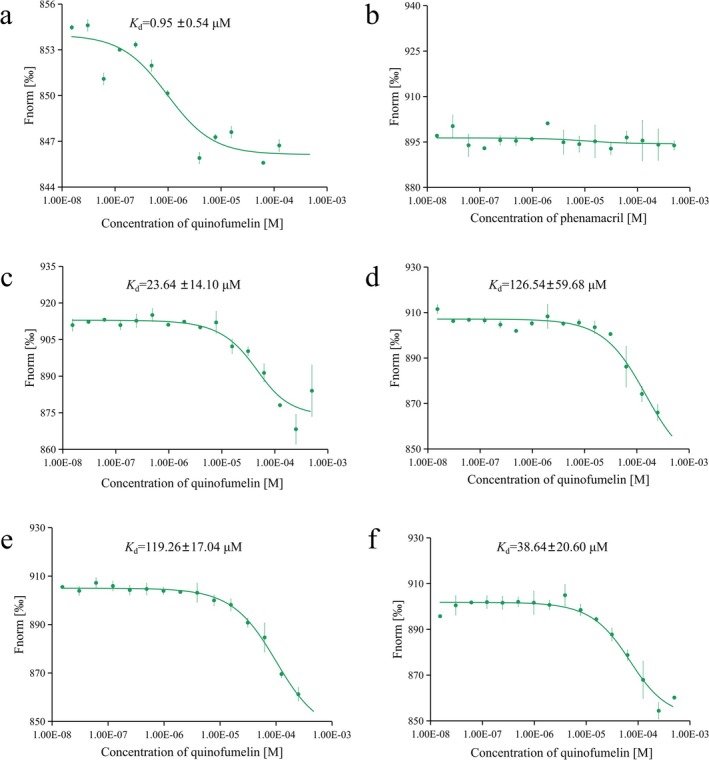
Microscale thermophoresis analysis for assessing the binding interaction between quinofumelin and FgDHODHII. (a) Binding affinity of quinofumelin to FgDHODHII wild‐type (WT) protein. (b) Binding affinity of phenamacril to FgDHODHII‐WT protein. (c–f) Binding affinity of quinofumelin to FgDHODHII‐D155T, FgDHODHII‐V179E, FgDHODHII‐V179D and FgDHODHII‐N281A proteins, respectively. The solid curves represent fitted data according to the Hill equation (*n* = 1). The error bars represent the standard error derived from triplicate measurements.

We further evaluated the binding affinity (*K*_d_ value) of quinofumelin for FgDHODHII using MST assays. The MST results demonstrated that FgDHODHII‐WT specifically bound to quinofumelin in vitro, with a *K*_d_ value of 0.95 ± 0.54 μM. In contrast, FgDHODHII‐D155T, FgDHODHII‐V179E, FgDHODHII‐V179D and FgDHODHII‐N281A exhibited *K*_d_ values of 23.64 ± 14.10, 126.54 ± 59.68, 119.26 ± 17.04 and 38.64 ± 20.60 μM, respectively (Figure 7), indicating significantly reduced binding affinities compared to FgDHODHII‐WT. No binding was observed with phenamacril, which does not exhibit cross‐resistance to quinofumelin. MST findings further confirm that the D155T, V179E, V179D and N281A mutations in FgDHODHII lead to decreased affinity for quinofumelin and support an inverse correlation between binding affinity and resistance level.

## 3 Discussion

Fusarium head blight (FHB), caused by the *F. graminearum* species complex, is a devastating fungal disease of global importance affecting crop yield and quality. In severe epidemic years, it can lead to substantial yield losses (Bai and Shaner 2004). Moreover, *F. graminearum* produces mycotoxins such as deoxynivalenol (DON) in infected grains, resulting in widespread mycotoxin contamination that compromises the safety of food and feed and poses serious risks to human and animal health (Hooft and Bureau 2021; Xiu et al. 2021; Zhang et al. 2026). Currently, chemical fungicides remain the primary strategy for controlling *F. graminearum* infection due to the limited availability of highly resistant wheat cultivars. However, prolonged use of single‐site fungicides has resulted in moderate to high‐resistance risks in *F. graminearum* against commonly used fungicides, including carbendazim and phenamacril (Duan et al. 2014).

In recent years, dihydroorotate dehydrogenase (DHODH) has emerged as a promising target in drug discovery. Inhibition of DHODH disrupts the de novo synthesis of DNA, RNA and glycoproteins, offering a means to regulate aberrant cellular metabolism and proliferation for therapeutic benefit (Reis et al. 2017; Singh et al. 2017). Leflunomide (metabolized to teriflunomide) and brequinar are classic DHODH inhibitors used for the clinical treatment of rheumatoid arthritis, cancer and multiple sclerosis in humans (Breedveld and Dayer 2000), and their co‐crystal structures with human DHODH have been resolved (PDB code: 1D3H) (Liu et al. 2000). In agriculture, F901318 (olorofim) exhibits potent activities against *Aspergillus* species and demonstrates broad applicability across fungal pathogens (Oliver et al. 2016). Additionally, ipflufenoquin has been shown to effectively control *Botrytis cinerea* strains resistant to SDHI, QoI and MBC fungicides (Choi et al. 2024). Quinofumelin, a quinoline fungicide, has been reported to exhibit strong antifungal activity against FHB pathogens (Xiu et al. 2021). Recent research by our group identified DHODHII as the molecular target of quinofumelin in *F. graminearum* (Xiu et al. 2025).

The investigation of resistance mechanisms is closely linked to mutations in target genes identified in resistant strains. A previous study has demonstrated that in vitro exposure of *Aspergillus fumigatus* to the agrochemical fungicide ipflufenoquin can lead to the selection of strains with cross‐resistance to olorofim (Rhijn et al. 2024). Notably, mutations such as H116R, G119C/S/A, L164P and V200E in DHODH have been associated with resistance to both ipflufenoquin and olorofim (Rhijn et al. 2024). Furthermore, under the selective pressure from high concentrations of olorofim, the G119C, G119S and G119A mutants increased in frequency and exhibited a stronger competitive advantage, whereas under high concentrations of ipflufenoquin the V200E mutant dominated the population (Rhijn et al. 2024). This indicates that different mutant genotypes exhibit varying degrees of fitness penalty under the selective pressure of fungicides. To further investigate the role of FgDHODHII in modulating sensitivity to quinofumelin, we constructed the three‐dimensional structure of the FgDHODHII protein using AlphaFold3 and performed molecular docking with quinofumelin using Autodock software. Subsequently, a conservation analysis of the docking sites was conducted across various pathogenic fungal species to identify potential resistance‐associated positions. The results showed that residues at positions 94, 155, 179 and 281 were highly conserved among 11 fungal species (Figure 1a). Moreover, the valine at position 200 in *A. fumigatus* corresponds to the valine at position 179 in *F. graminearum*. Accordingly, we generated *F. graminearum* mutants carrying A94V, D155T, V179E, V179D or N281A mutations in FgDHODHII by the homologous recombination strategy (Liu et al. 2013; Malardier et al. 1989). The sensitivity of the wild‐type strain PH‐1 and site‐directed mutants to quinofumelin was then assessed using mycelial growth inhibition method. The results showed that FgDHODHII‐A94V remained sensitive to quinofumelin, FgDHODHII‐D155T exhibited low‐level resistance, FgDHODHII‐N281A displayed moderate‐level resistance, while FgDHODHII‐V179E and FgDHODHII‐V179D showed high‐level resistance (Figure 2a). These findings indicate that DHODHII serves as the action target of quinofumelin against plant‐pathogenic fungi. This is consistent with the result that the V200E mutation in *A. fumigatus* confers high‐resistance to olorofim and ipflufenoquin (Rhijn et al. 2024). Recovery experiments indicated that quinofumelin might inhibit the biosynthesis of essential cellular components that can be exogenously supplemented from the culture medium. Despite differences in sensitivity among the mutants when exposed to 10 μg/mL quinofumelin on PDA plates, only FgDHODHII‐V179E and FgDHODHII‐V179D exhibited partial growth. Importantly, exogenous supplementation with 50 μg/mL UMP, uridine or uracil effectively restored mycelial growth in both PH‐1 and all mutants (Figure 2b). These findings further confirm that quinofumelin effectively inhibits orotate formation in the pyrimidine biosynthesis pathway.

To further evaluate the risk of *F. graminearum* developing resistance to quinofumelin, we measured mycelial growth rate, sporulation capacity and virulence of site‐directed mutants. The findings showed no significant difference in mycelial growth rate among all site‐directed mutants compared with PH‐1 (Figure 2c). Additionally, the sporulation capacity and virulence of FgDHODHII‐A94V were not significantly different from those of the sensitive strain PH‐1. However, these traits were significantly reduced in FgDHODHII‐D155T, FgDHODHII‐V179E, FgDHODHII‐V179D and FgDHODHII‐N281A (Figure 2d; Figure 3). These findings suggest that quinofumelin resistance suffers a fitness penalty. Understanding cross‐resistance patterns is crucial for formulating effective resistance management strategies. The results indicate that quinofumelin shows cross‐resistance only with ipflufenoquin and exhibits no cross‐resistance to other commonly used fungicides (phenamacril, carbendazim, tebuconazole, pydiflumetofen, prothioconazole, fluopyram, benzovindiflupyr, pyraclostrobin and azoxystrobin) (Figure 4). These findings indicate that *F. graminearum* poses a medium‐to‐high resistance risk to quinofumelin, suggesting that ipflufenoquin may share a similar mode of action. Importantly, quinofumelin showed no cross‐resistance with other commonly used fungicides, supporting its potential suitability for integrated resistance management of FHB.

Molecular docking is a computational approach used to predict the preferred binding orientation between a ligand and its target receptor. Here, molecular docking results revealed a strong interaction between FgDHODHII‐WT and quinofumelin, with a binding energy of −6.54 kcal/mol. The A94V mutation did not substantially affect this interaction. In contrast, the D155T, V179E, V179D and N281A mutations altered the binding mode of quinofumelin, resulting in reduced binding affinities, with corresponding energies of −5.96, −5.47, −5.35 and −6.02 kcal/mol, respectively (Figure 5). These calculated binding energies negatively correlated with the resistance levels observed for the corresponding mutants. Molecular dynamics (MD) simulations further demonstrated that the D155T, V179E, V179D and N281A mutations impaired binding stability with quinofumelin, disrupted key interactions within the binding pocket, and reduced MM/PBSA‐derived binding free energies (−33.98, −29.49, −28.65 and −31.38 kcal/mol, respectively), consistent with their elevated resistance levels (Figure 6; Table 3). These findings were further supported by MST assays (Figure 7). Collectively, these results provide compelling evidence that the D155T, V179E, V179D and N281A mutations in FgDHODHII confer resistance to quinofumelin. This study represents the first report elucidating the molecular mechanism of quinofumelin resistance in *F. graminearum*.

## 4 Experimental Procedures

### 4.1 Strains, Fungicides and Culture Conditions

*Fusarium graminearum* PH‐1 and the PH‐1 deletion mutants ΔFgDHODHII were maintained in the Fungicide Biology Laboratory, Nanjing Agricultural University.

Quinofumelin (a.i. 95%), phenamacril (a.i. 95%, CAS: 39491‐78‐6), tebuconazole (a.i. 95%, CAS: 107534‐96‐3), pydiflumetofen (a.i. 98%, CAS: 1228284‐64‐7), prothioconazole (a.i. 98%, CAS: 178928‐70‐6), fluopyram (a.i. 96%, CAS: 658066‐35‐4), carbendazim (a.i. 98%, CAS: 10605‐21‐7), benzovindiflupyr (a.i. 96%, CAS: 1072957‐71‐1), ipflufenoquin (a.i. 96%, CAS: 1314008‐27‐9), pyraclostrobin (a.i. 97.5%, CAS: 175013‐18‐0) and azoxystrobin (a.i. 98%, CAS: 131860‐33‐8) were kindly provided by Mitsui Chemicals Co. Ltd., Jiangsu Pesticide Research Institute Co. Ltd., Hubei Kangbaotai Fine Chemical Co. Ltd., Syngenta Investment Co. Ltd., Bayer Co. Ltd. (China), Bayer Co. Ltd. (China), Jiangyin Pesticide Factory Co. Ltd., Hebei Saifeng Biotechnology Co. Ltd., Mitsui Chemicals Co. Ltd., Jiangsu Yangnong Chemical Co. Ltd., Shaanxi Sunger Road Bio‐science Co. Ltd., respectively.

Dihydroorotate hydrogenase, UMP, undine and uracil were purchased from Shanghai Macklin Biochemical Technology Co. Ltd. and dissolved in water for storage and usage.

PDA (200 g potato, 20 g dextrose and 16 g agar powder in 1 L water), YBA (10 g yeast extract, 10 g peptone, 20 g sodium acetate anhydrous and 15 g agar powder per in 1 L water) and AEA (5 g yeast extract, 6 g NaNO_3_, 1.5 g KH_2_PO_4_, 0.5 g KCl, 0.51 g MgSO_4_·7H_2_O, 16 g agar powder and 20 mL glycerol per in 1 L water) were employed for regular mycelial growth and fungicide sensitivity testing cultures. Mung bean broth (MBB) (30 g boiled mung beans in 1 L water) was employed for spore production. Rich medium (RM) (1 g casein hydrolysate, 1 g yeast extract, 342.29 g sucrose and 16 g agar powder in 1 L water) and SRM medium (1 g casein hydrolysate, 1 g yeast extract, 342.29 g sucrose and 10 g agarose in 1 L water) were used for protoplast transformation. All media were sterilized by autoclaving at 120°C for 20 min.

### 4.2 Sequence Analysis of DHODHII

To investigate the potential target of *F. graminearum* resistance to quinofumelin, we performed a sequence alignment analysis on the available DHODHII proteins of various fungal species. Specifically, we aligned the protein sequences of DHODHII from *F. graminearum* (XP_011328027), *F. pseudograminearum* (XP_009255676), *F. oxysporum* (KAG7415722), *A. fumigatus* (KAK9596360), *A. nidulans* (AAA86932), *B. cinerea* (XP_024553432), *Pseudocercospora fuligena* (KAF7189329), *S. sclerotiorum* (XP_001585164), *Corynespora cassiicola* (PSN63766), *Rhizoctonia solani* (EUC60862) and *Alternaria alternata* (XP_018389332) using BioEdit software.

### 4.3 Generation of Vectors of Site‐Directed Mutagenesis

To confirm whether the resistance of *F. graminearum* to quinofumelin is attributed to the key mutations of A94V, D155T, V179E, V179D and N281A in DHODHII, the homologous double‐exchange vectors of the mutants harbouring the A94V, D155T, V179E, V179D and N281A mutations in FgDHODHII were constructed. These mutants of FgDHODHII were generated through protoplast transformation, as previously described with some modifications in the transformation procedures (Liu et al. 2013; Malardier et al. 1989). The left‐side, middle and right‐side fragments of the target gene were amplified with a targeted base mismatch introduced between primers 94‐V‐F/R, 155‐T‐F/R, 179‐E‐F/R, 179‐D‐F/R or 281‐A‐F/R (all primers are shown in Table 4). Finally, the left‐side, middle and right‐side fragments were fused at a 1:3:1 ratio. The complete vector was obtained by amplifying the fused product with primers FGSG_09678‐SB‐F and FGSG_09678‐XB‐R.

**TABLE 4:** Primers used in this study.

Primer	Sequence (5′–3′)	Use
FGSG_09678‐SB‐F	GGAGGTTGCATGTCAAGCTT	Amplification of the left flank of the site‐direct vector
FGSG_09678‐SB‐R	ATGGCTGGGAAGTAATGCTG	
FGSG_09678‐XB‐F	GACATGCTATAGCAAGCAAC	Amplification of the right flank of a site‐direct vector
FGSG_09678‐XB‐R	CTAACGAGCAACCGTCAGCA	
94‐V‐F	ATGCCGAGGACGTCCACCAT	Primers containing mismatched bases (the mutant harbouring the A94V mutation in FgDHODHII)
94‐V‐R	ATGGTGGACGTCCTCGGCAT	
155‐T‐F	GTTGTTTACCCTTGGAGCTG	Primers containing mismatched bases (the mutant harbouring the D155T mutation in FgDHODHII)
155‐T‐R	CAGCTCCAAGGGTAAACAAC	
179‐E‐F	AGCCTCGAGAGTTCCGCGTT	Primers containing mismatched bases (the mutant harbouring the V179E mutation in FgDHODHII)
179‐E‐R	AACGCGGAACTCTCGAGGCT	
179‐D‐F	AGCCTCGAGACTTCCGCGTT	Primers containing mismatched bases (the mutant harbouring the V179D mutation in FgDHODHII)
179‐D‐R	AACGCGGAAGTCTCGAGGCT	
281‐A‐F	GCTGGTCGTGGCCGTCAGCA	Primers containing mismatched bases (the mutant harbouring the N281A mutation in FgDHODHII)
281‐A‐R	TGCTGACGGCCACGACCAGC	
FGSG_09678‐F	CAGCATTACTTCCCAGCCAT	Amplification of a fragments containing site‐directed mutagenesis for sequencing
FGSG_09678‐R	GTTGCTTGCTATAGCATGTC	

### 4.4 Protoplast Transformation and Transformants Validation

ΔFgDHODHII protoplasts were prepared under the condition of adding uridine, and transformation analysis was performed according to previously described methods (Liu et al. 2013; Malardier et al. 1989). The site‐directed fragments of the mutants harbouring the A94V, D155T, V179E, V179D and N281A mutations in FgDHODHII were used to replace the double‐screened genes in the PH‐1 deletion mutants ΔFgDHODHII. The putative transformants were selected on PDA plates containing 100 μg/mL 5‐fluoro‐2‐deoxyuridine (F2dU). Subsequently, the transformants were amplified and sequenced with primers FGSG_09678‐F and FGSG_09678‐R to confirm the successful replacement.

### 4.5 The Sensitivity of Site‐Directed Mutants to Quinofumelin

The sensitivity of PH‐1 and FgDHODHII‐A94V, FgDHODHII‐D155T, FgDHODHII‐V179E, FgDHODHII‐V179D and FgDHODHII‐N281A to quinofumelin was determined by the mycelial growth inhibition method as previously described (Duan et al. 2013). Fresh mycelial plugs (5 mm in diameter) were cut from the edge of a 3‐day‐old colony and transferred onto PDA plates containing varying concentrations of quinofumelin. For PH‐1 and FgDHODHII‐A94V, the concentrations were 0, 0.01, 0.02, 0.04, 0.08 and 0.16 μg/mL of quinofumelin; for FgDHODHII‐D155T, FgDHODHII‐V179E, FgDHODHII‐V179D and FgDHODHII‐N281A, the concentrations were 0, 0.1, 0.5, 2.5, 12.5 and 62.5 μg/mL. After the plates were incubated at 25°C for 3 days, the colony diameter was measured using the criss‐cross method. The EC_50_ (50% effective concentration) was calculated using DPS software (v. 7.05).

### 4.6 Recovery Assay of Mycelial Growth Inhibition by Quinofumelin in *F. graminearum*

PH‐1 and FgDHODHII‐A94V, FgDHODHII‐D155T, FgDHODHII‐V179E, FgDHODHII‐V179D and FgDHODHII‐N281A mutants were precultured on a PDA plate to form mycelial colonies. Fresh mycelial plugs were cut from the edge of a 3‐day‐old colony and inoculated onto PDA plates containing 10 μg/mL quinofumelin and 50 μg/mL dihydroorotate, UMP, uridine or uracil. The inoculated plates were subsequently incubated at 25°C for 3 days, then observed for the growth of mycelial colonies. Each concentration was repeated three times, and the experiment was repeated three times.

### 4.7 Determination of Mycelial Growth Rate Assay

Mycelial growth rate was determined by inoculating 5‐mm‐diameter fresh plugs cut from the colony onto PDA plates and incubating them at 25°C in the dark for 3 days; colony diameter was then measured with the criss‐cross method.

### 4.8 Sporulation Assay

Five mycelial plugs from the above‐mentioned colony were added to each 30 mL of MBB medium. After 3 days of shaking culture at 25°C and 175 rpm, mycelial plugs were filtered through three layers of filter paper. The filtrate was centrifuged at 5000 rpm for 5 min, discarded the supernatant and 2 mL double‐distilled water was added to resuspend the spores. Finally, spore concentration was determined under a microscope using the haemocytometer.

### 4.9 Virulence Assay

In order to explore the effect of the site‐directed mutants on the virulence of wheat, we conducted infection experiments on wheat coleoptiles and flowering wheat heads. When the length of the wheat coleoptile was about 2 cm, the tip was removed with a disinfected scissors, and 2 μL of conidial suspension with a concentration of 1 × 10^6^/mL was inoculated into the tip of the coleoptile with a pipette. The wheat was placed in a 25°C greenhouse for 7 days, and the lesion length of the wheat roots was measured and photographed. Wheat heads at flowering stage were selected in the experimental field, and 10 μL of conidial suspension (1 × 10^6^/mL) was inoculated into the heads with a pipette and marked with a black marker. Each strain was inoculated onto 30 wheat heads. After 21 days, the incidence of disease on the wheat heads was observed and photographed. Each treatment was set up with 30 plants.

### 4.10 Cross‐Resistance Assay

The cross‐resistance between quinofumelin and other fungicides in *F. graminearum* was determined using PH‐1 and the site‐directed mutants. Fresh mycelial plugs were cut from the edge of a 3‐day‐old colony of PH‐1 and site‐directed mutants, and inoculated on PDA plates containing different concentrations of quinofumelin, phenamacril, carbendazim, tebuconazole, prothioconazole and ipflufenoquin, YBA plates of benzovindiflupyr, pydiflumetofen and fluopyram, and AEA plates of pyraclostrobin and azoxystrobin (Table 5). After incubation at 25°C for 3 days, colony diameters were measured, and EC_50_ values for various fungicides were calculated. The Spearman correlation test was employed to determine the presence of cross‐resistance between quinofumelin and other fungicides.

**TABLE 5:** Fungicide concentrations for cross‐resistance assay.

Fungicide	Fungicide concentrations (μg/mL)	
	Wild‐type PH‐1 and FgDHODHII‐A94V	Other site‐directed mutants
Quinofumelin	0, 0.01, 0.02, 0.04, 0.08, 0.16	0, 0.1, 0.5, 2.5, 12.5, 62.5
Phenamacril	0, 0.0625, 0.125, 0.25, 0.5, 1	
Carbendazim	0, 0.0625, 0.125, 0.25, 0.5, 1	
Tebuconazole	0, 0.0625, 0.125, 0.25, 0.5, 1	
Pydiflumetofen	0, 0.001, 0.002, 0.004, 0.008, 0.016	
Prothioconazole	0, 0.25, 0.5, 1, 2, 4	
Fluopyram	0, 0.01, 0.02, 0.04, 0.08, 0.16	
Benzovindiflupyr	0, 0.0001, 0.001, 0.01, 0.1, 1	
Ipflufenoquin	0, 0.0625, 0.25, 1, 4, 16	0, 0.78125, 3.125, 12.5, 50, 200
Pyraclostrobin	0, 0.003125, 0.0125, 0.05, 0.2, 0.8	
Azoxystrobin	0, 0.001, 0.01, 0.1, 1, 10	

### 4.11 Molecular Docking

The three‐dimensional structures of quinofumelin and six types of DHODHII proteins from *F. graminearum* were subjected to molecular docking analysis to evaluate their respective binding affinities. The amino acid sequences of FgDHODHII and the mutants harbouring the A94V, D155T, V179E, V179D and N281A mutations in FgDHODHII were submitted to the AlphaFold Server (http://golgi.sandbox.google.com) for obtaining three‐dimensional protein models. The three‐dimensional structure of quinofumelin (PubChem CID: 23160856) used for docking was obtained from the PubChem database. AutoDockTools v. 1.5.7 was employed for conducting the molecular docking simulations. In the docking studies, binding energy values given by AutoDockTools software were used to assess the binding affinity between the target protein and quinofumelin. The smaller binding energy values show a stronger binding affinity between the protein and the molecule. The resulting docking outcomes were visualized using PyMOL software v. 2.6.

### 4.12 Molecular Dynamics Simulations

MD simulations were conducted to analyse the docked structures of FgDHODHII with quinofumelin using the GROMACS2025 software package (Abraham et al. 2015). The topology and coordinate files for the compounds were generated using the AMBER 14SB force field and converted into the Gromacs topology and coordinate files using pdb2gmx and ACPYPE program (Maier et al. 2015). The topology and coordinate files of the protein and ligand were merged and immersed into a dodecahedron periodic box with the SPC216 explicit solvation model in a rectangular box and then neutralized by Na^+^ and Cl^−^ counterions. Energy minimization was performed using the steepest descent method, followed by position‐restrained equilibration in NVT and NPT ensembles at 298 K and 1.0 bar. The MD simulations generated a 200 ns trajectory using the leapfrog integrator with a 2 fs time step. The temperature and pressure were maintained by the modified Berendsen thermostat and Parrinello‐Rahman barostat (Amadei et al. 1993; Bussi et al. 2007). All bonds involving hydrogen atoms were constrained by the LINCS algorithm. The coordinates and energy of each atom were stored every 10 ps for further analysis. To evaluate the conformational stability and flexibility of the complex in the solvated environment, RMSD and RMSF were computed throughout the simulation trajectory.

### 4.13 MM/PBSA‐Trajectory Analysis

MM/PBSA‐trajectory analysis was performed using the molecular mechanics Poisson‐Boltzmann surface area (MM‐PBSA) method, an established computational approach widely employed to estimate the binding free energy of protein–ligand complexes. The binding free energy is calculated according to the following equation (Genheden and Ryde 2015):

$$\mathrm{\Delta G}\;\left(\text{Binding}\right)=\mathrm{\Delta G}\;\left(\text{Complex}\right)-\left(\mathrm{\Delta G}\;\text{Protein}+\mathrm{\Delta G}\;\text{Ligand}\right),$$

where ΔG (Complex) denotes the total free energy of the bound complex, and ΔG Protein and ΔG Ligand represent the free energies of the isolated protein and ligand, respectively.

### 4.14 MST Assay

Binding reactions of recombinant FgDHODHII‐GFP with quinofumelin were measured by MST in a Monolith NT.Label Free (Nano Temper Technologies) instrument. The GFP fusion protein was extracted from mycelia by phosphate‐buffered saline (PBS). Labelled proteins were used at a final concentration of 50 nM. A range of concentrations of quinofumelin in PBS were incubated with labelled protein (1:1, v/v). The samples were loaded into the NT.Label Free standard capillaries and performed with 80% (Auto‐detect) Nano‐Blue. Monolith NT.115 software was used to evaluate the binding affinity between FgDHODHII and quinofumelin using the *K*_d_ values and the signal to noise ratio, and the results were combined and analysed using Mo. Affinity Analysis v. 2.3 software.

### 4.15 Statistical Analysis

All data in the study were analysed by DPS software (v. 7.05). For the calculation of EC_50_ value, logarithmic conversion of fungicide concentration values and probability value conversion of growth inhibition percentages were performed to facilitate subsequent calculations. Results from the enzyme activity, mycelial growth rate, sporulation capability and virulence were analysed by the one‐way ANOVA, followed by Fisher's least significant difference (LSD) test with a level of significance of 5% (*p* < 0.05).

## Author Contributions

**Xiaoru Yin:** data curation, formal analysis, investigation, methodology, project administration, supervision, validation, visualization, writing – original draft. **Xinlong Gao:** data curation, investigation. **Xudong Liu:** software, visualization. **Ziyang Zhang:** investigation, methodology. **Qian Xiu:** investigation, methodology. **Fuhao Ren:** investigation, methodology. **Jie Zhang:** methodology. **Mingguo Zhou:** project administration. **Yabing Duan:** project administration, resources, writing – review and editing.

## Funding

This work was supported by the National Key Research and Development Program of China (2022YFD1400100), Joint Research Program of State Key Laboratory of Agricultural and Forestry Biosecurity (SKLJRP2510) and the National Natural Science Foundation of China (32072448).

## Conflicts of Interest

The authors declare no conflicts of interest.

## Data Availability

The data that support the findings of this study are available on request from the corresponding author. The data are not publicly available due to privacy or ethical restrictions.
